# Hyperhydration to Improve Kidney Outcomes in Children with Shiga Toxin-Producing *E. coli* Infection: a multinational embedded cluster crossover randomized trial (the HIKO STEC trial)

**DOI:** 10.1186/s13063-023-07379-w

**Published:** 2023-05-27

**Authors:** Stephen B. Freedman, David Schnadower, Myka Estes, T. Charles Casper, Stuart L. Goldstein, Silviu Grisaru, Andrew T. Pavia, Benjamin S. Wilfond, Melissa Metheney, Kadyn Kimball, Phillip I. Tarr, On behalf of the Hyperhydration to Improve Kidney Outcomes in children with Shiga Toxin-producing E. Coli infection (HIKO-STEC) Study Team

**Affiliations:** 1 Sections of Pediatric Emergency Medicine and Gastroenterology, Departments of Pediatrics and Emergency Medicine, Alberta Childrens Hospital, Alberta Childrens Hospital Research Institute, Cumming School of Medicine, University of Calgary, Calgary, AB Canada; 2 Division of Emergency Medicine, Cincinnati Children, s Hospital Medical Center and Department of Pediatrics University of Cincinnati College of Medicine, Cincinnati, OH USA; 3 Departments of Pediatrics and Emergency Medicine, Alberta Children’s Hospital, Cumming School of Medicine, University of Calgary, Calgary, AB Canada; 4 Department of Pediatrics, University of Utah, Salt Lake City, UT USA; 5 Center for Acute Care Nephrology, Cincinnati Children, s Hospital Medical Center and Department of Pediatrics University of Cincinnati College of Medicine, Cincinnati, OH USA; 6 Section of Nephrology, Department of Pediatrics, Alberta Children, s Hospital, Cumming School of Medicine, University of Calgary, Calgary, AB Canada; 7 Division of Pediatric Infectious Diseases, Department of Pediatrics and Internal Medicine, University of Utah, Salt Lake City, UT USA; 8 Divisions of Bioethics and Palliative Care and Pulmonary and Sleep Medicine, Department of Pediatrics and Department of Bioethics and Humanities, University of Washington School of Medicine, Seattle, WA USA; 9 Division of Gastroenterology, Hepatology, & Nutrition, Department of Pediatrics, Washington University in St. Louis School of Medicine, St. Louis, MO USA

**Keywords:** Shiga toxin-producing E. coli (STEC), Hemolytic uremic syndrome (HUS), Hyperhydration, Euvolemia, Children, Gastroenteritis, Bloody diarrhea, Emergency department, Acute kidney injury, Hospitalization

## Abstract

**Background:**

Shiga toxin-producing *E. coli* (STEC) infections affect children and adults worldwide, and treatment remain solely supportive. Up to 15–20% of children infected by high-risk STEC (i.e., *E. coli* that produce Shiga toxin 2) develop hemolytic anemia, thrombocytopenia, and kidney failure (i.e., hemolytic uremic syndrome (HUS)), over half of whom require acute dialysis and 3% die. Although no therapy is widely accepted as being able to prevent the development of HUS and its complications, several observational studies suggest that intravascular volume expansion (hyperhydration) may prevent end organ damage. A randomized trial is needed to confirm or refute this hypothesis.

**Methods:**

We will conduct a pragmatic, embedded, cluster-randomized, crossover trial in 26 pediatric institutions to determine if hyperhydration, compared to conservative fluid management, improves outcomes in 1040 children with high-risk STEC infections. The primary outcome is major adverse kidney events within 30 days (MAKE30), a composite measure that includes death, initiation of new renal replacement therapy, or persistent kidney dysfunction. Secondary outcomes include life-threatening, extrarenal complications, and development of HUS. Pathway eligible children will be treated per institutional allocation to each pathway. In the hyperhydration pathway, all eligible children are hospitalized and administered 200% maintenance balanced crystalloid fluids up to targets of 10% weight gain and 20% reduction in hematocrit. Sites in the conservative fluid management pathway manage children as in- or outpatients, based on clinician preference, with the pathway focused on close laboratory monitoring, and maintenance of euvolemia. Based on historical data, we estimate that 10% of children in our conservative fluid management pathway will experience the primary outcome. With 26 clusters enrolling a mean of 40 patients each with an intraclass correlation coefficient of 0.11, we will have 90% power to detect a 5% absolute risk reduction.

**Discussion:**

HUS is a devastating illness with no treatment options. This pragmatic study will determine if hyperhydration can reduce morbidity associated with HUS in children with high-risk STEC infection.

**Trial registration:**

ClinicalTrials.gov NCT05219110. Registered on February 1, 2022.

## Administrative information

Note: the numbers in curly brackets in this protocol refer to SPIRIT checklist item numbers. The order of the items has been modified to group similar items (see http://www.equator-network.org/reporting-guidelines/spirit-2013-statement-defining-standard-protocol-items-for-clinical-trials/).

**Table:** 

Title {1}	**Hyperhydration to Improve Kidney Outcomes in Children with Shiga Toxin-Producing ** ***E. coli*** ** Infection: A Multinational Embedded Cluster Crossover Randomized Trial (The HIKO STEC trial)**
Trial registration {2a and 2b}.	ClinicalTrials.gov: NCT05219110
Protocol version {3}	V3.6 02/06/2023
Funding {4}	NIAID R01 AI165327 CIHR # 469,594 The Pediatric Emergency Care Applied Research Network is supported by the Health Resources and Services Administration, Maternal and Child Health Bureau, Emergency Medical Services for Children Program through the following cooperative agreements: U03MC00001, U03MC00003, U03MC00006, U03MC00007, U03MC00008, U03MC22684, and U03MC22685. Alberta Children’s Hospital Foundation Professorship in Child Health and Wellness
Author details {5a}	**Stephen B. Freedman**: Sections of Pediatric Emergency Medicine and Gastroenterology, Departments of Pediatrics and Emergency Medicine, Alberta Children's Hospital, Alberta Children's Hospital Research Institute, Cumming School of Medicine, University of Calgary, Calgary, AB, Canada **David Schnadower**: Division of Emergency Medicine, Cincinnati Children’s Hospital Medical Center and Department of Pediatrics University of Cincinnati College of Medicine, Cincinnati, OH, USA **Myka Estes**: Departments of Pediatrics and Emergency Medicine, Alberta Children's Hospital, Cumming School of Medicine, University of Calgary, Calgary, AB, Canada **T. Charles Casper**: Department of Pediatrics, University of Utah, Salt Lake City, UT, USA **Stuart L. Goldstein**: Center for Acute Care Nephrology, Cincinnati Children’s Hospital Medical Center and Department of Pediatrics University of Cincinnati College of Medicine, Cincinnati, OH, USA **Silviu Grisaru**: Section of Nephrology, Department of Pediatrics, Alberta Children's Hospital, Cumming School of Medicine, University of Calgary, Calgary, AB, Canada **Andrew T. Pavia**: Division of Pediatric Infectious Diseases, Department of Pediatrics and Internal Medicine, University of Utah, Salt Lake City, UT, USA **Benjamin S. Wilfond**: Divisions of Bioethics and Palliative Care and Pulmonary and Sleep Medicine, Department of Pediatrics and Department of Bioethics and Humanities, University of Washington School of Medicine **Melissa Metheney**: Department of Pediatrics, University of Utah, Salt Lake City, UT, USA **Kadyn Kimball**: Department of Pediatrics, University of Utah, Salt Lake City, UT, USA **Phillip I. Tarr:** Division of Gastroenterology, Hepatology, & Nutrition, Department of Pediatrics, Washington University in St. Louis School of Medicine, St. Louis, MO, USA
Name and contact information for the trial sponsor {5b}	Sponsor: University of Calgary Contact Info: Stephen B. Freedman, MDCM, MSc: 403–955-7740
Role of sponsor {5c}	The sponsor will not participate in study design, collection, management, analysis, data interpretation or in the writing of the report, or the decision to submit the report for publication.

## Introduction

### Background and rationale {6a}

Globally, there are over 2.8 million [1] cases of Shiga toxin-producing *E. coli* (STEC) infection each year. In 2012, the Centers for Disease Control and Prevention estimated that 96,534 high-risk ((i.e., Shiga toxin 2-producing (Stx 2)) STEC infections occur yearly in the US [2, 3]. Over 60% of these infections occur in children, half of whom are under 5 years old [2, 4]. Hemolytic uremic syndrome (HUS, the triad of hemolytic anemia, thrombocytopenia, and kidney failure) is the most serious potential complication of STEC infections [5], and, in North America, *E. coli* O157:H7 is the most common cause of this post-diarrheal microangiopathic disorder.

Most STEC-infected children present for care when bloody diarrhea prompts medical attention; 15–20% of children infected with high-risk STEC develop HUS, and this complication ensues a median of ~ 7.5 days following the onset of diarrhea [6]. In recent years, mortality of HUS remains about 3% [7]. Up to half of survivors have chronic kidney disease [8]. HUS is believed to be a consequence of a cascade triggered by brief circulation of Stx early in the illness; thus, to prevent HUS or diminish its severity, interventions early in illness are needed.

Over four decades of research on STEC have produced elegant cellular and pathogen genomic insights but have not generated any interventions to reduce or avoid major complications including acute kidney injury (AKI). Narcotics [9–12], antibiotics [7, 13–15], antimotility agents [9, 10], and non-steroidal anti-inflammatory drugs [14] are either ineffective or predispose to poor outcomes. In the absence of effective treatments, there is evidence of considerable practice variation [16, 17]. A multicenter study showed that 59% of STEC-infected children presenting to pediatric emergency departments were directly hospitalized, but of those managed as outpatients, 17% returned to hospital with HUS [16]. This reflects the wide spectrum of care provided to STEC-infected children with treatments varying from a “wait and see” approach (i.e., children managed at home and instructed to seek care when complications ensue) to “admit all” for high-volume hyperhydration (see below). This variation reflects the lack of evidence to guide care, and the limited awareness of front-line clinicians (i.e., family physicians, pediatricians, emergency physicians) regarding the natural history of STEC infection [17].

Observational studies have shown that early high-volume intravenous fluid administration (hyperhydration) is associated with reduced severity of kidney injury and may be effective in mitigating or preventing complications [18–20]. Even though vascular injury is underway by the time children present with STEC-induced diarrhea [21, 21–23], we hypothesize that there is an opportunity to reduce kidney damage and maintain kidney function through intravascular volume expansion in children who present for care before HUS ensues [24, 25]. This hypothesis is generated from three complementary sets of data: the poor outcomes associated with STEC infections in children who have relative hemoconcentration (and other signs of volume contraction) when they present with HUS, the partial success of aggressive intravenous volume expansion at the point of diagnosis with HUS, and improved outcomes in observational studies where large volumes of isotonic intravenous fluids were administered before the development of HUS.

### Volume contraction when HUS is diagnosed is a risk for poor outcomes [26]

Multiple series of children with diarrhea-associated HUS demonstrate that hemoconcentration and/or dehydration at admission (i.e., when HUS is diagnosed) have worse outcomes [7, 27–31].

### Protocolized volume expansion at diagnosis of HUS might reduce the severity of the subsequent clinical course

Based on data suggesting that volume contraction at presentation with HUS is a risk factor for poor outcomes, Ardissino et al. developed a systematic approach to the management of newly presenting children with HUS. In a study of 76 children using a pre-post design and a “treat-to-target” approach with a goal of 10% body weight increase from baseline, these authors report that renal replacement therapy (RRT) use decreased from 58 to 26% [18]. A similarly designed study in Argentina demonstrated that 16 children consecutively presenting with HUS who received 27 mL/kg over a 3-h period had greater urine output and were less likely to require RRT than 19 historical controls who were fluid restricted upon admission.

### Pre-HUS volume expansion is associated with less severe episodes of HUS

In a prospective, single-center study of 29 children with HUS [20], those who developed anuria received less intravenous sodium and fluid volumes during the pre-HUS period. Similar findings emerged from in an 11-center prospective cohort study of 50 children with HUS [19]—those who received no intravenous fluids within 4 days of diarrhea onset (i.e., before they developed HUS) were 1.6 times more likely to develop oligoanuric renal failure.

Additional data from the diarrhea phase pre-HUS suggest the need for, and feasibility of, volume expansion early in illness, to reduce the severity of HUS. McKee et al. studied 866 children infected with STEC, who did not have HUS at initial presentation in a network of 38 North American pediatric emergency departments [16]. In regression analysis, the development of HUS was associated with higher initial hematocrit values and the delayed receipt of intravenous fluids.

A meta-analysis of eight studies and 1511 children [26] concluded that intravenous fluid administration up to and including the day of HUS diagnosis was associated with a reduced need for RRT [26]. Moreover, relative hemoconcentration was associated with the development of oligoanuric renal failure and death [26]. In addition, compared to euvolemic patients, clinically dehydrated patients at HUS diagnosis have an increased risk of death [26].

The feasibility of reducing the anuria rate was suggested in a study of 36 STEC-infected children who were admitted to hospital before they developed HUS [13], most of whom received aggressive parenteral volume expansion. Only 11 (31%) of these 36 patients experienced anuria, a rate much lower than that in other recent series, where typically 50 to 60% [16, 32–34] require RRT. This study complements observational data supporting the benefit of volume expansion in the pre-HUS phase [13, 19, 20, 35] and lends support to the hypothesis that RRT rates can be reduced.

These multiple lines of evidence suggest the potential benefits of intravascular volume expansion in STEC-infected children. However, the strength of the conclusions that can be drawn and the impact on clinical care are limited by heterogeneity of design, small numbers, lack of clear protocols, unmeasured confounders, and difficulty differentiating causality from association. Our proposed study aims to resolve this uncertainty by comparing protocolized management of hyperhydration versus conservative fluid management.

### Objectives {7}

Our *primary objective* is to determine whether children diagnosed with a high-risk STEC infection who receive early intravenous fluid administration targeting intravascular volume expansion (i.e., hyperhydration) experience better kidney outcomes as defined by major adverse kidney events within 30 days (MAKE30) compared to children treated with a conservative fluid management approach.

Our *secondary objectives* are to determine whether there are differences in (1) the frequency of life-threatening, extrarenal complications of HUS and (2) the proportion that develops HUS of any severity between participants allocated to receive hyperhydration, compared to those allocated to receive conservative fluid management.

Our *exploratory/tertiary objectives* are to further our understanding of the pathophysiology of HUS and identify predictors of HUS.

### Trial design {8}

This is a phase III, multinational, multicenter, embedded, cluster-randomized, crossover trial comparing hyperhydration versus conservative fluid management of children with high-risk STEC infections.

## Methods: participants, interventions, and outcomes

### Study setting {9}

**Fig. 1:**
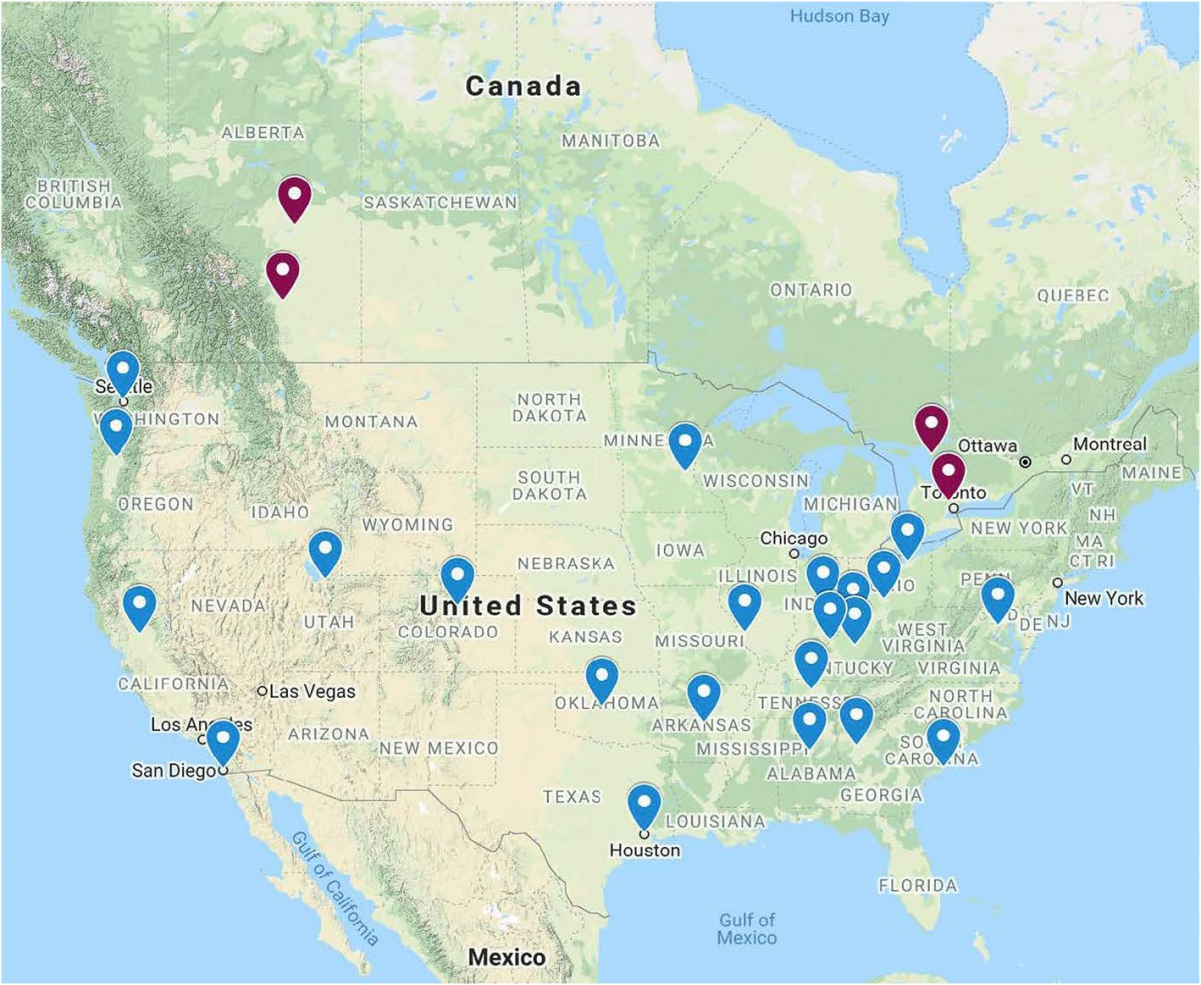
Location of 26 participating tertiary care academic pediatric institutions

Twenty-six tertiary care academic pediatric institutions located in the United States and Canada, and their patient catchment regions (Fig. 1).

### Eligibility criteria {10}

#### Screening criteria

Participating sites will establish a systematic process to identify all children with high-risk STEC infections within their institution’s catchment area. The process varies by site but includes the creation of electronic dashboards that identify all stool specimens that are positive for an STEC pathogen and the screening of all admission and discharge diagnoses. In addition, as many sites serve as referral institutions, any patient identified as likely having a high-risk STEC infection (defined below) within 24 h of result notification will be screened for eligibility. This time limit ensures that the participating site’s allocated treatment pathway can be initiated early in the disease course. Patients identified only because their condition deteriorated and were deemed to require transfer to a participating hospital for tertiary level care were ineligible as their inclusion would bias our study towards the null hypothesis as most such children will experience the primary outcome shortly after enrollment and would not have received the allocated pathway prior to transfer. Once screened and the clinical care pathway initiated, patients could be approached for study participation (which is limited to data and biospecimen collection as the clinical care pathways are embedded into routine care at study sites) up to 7 days following identification.

#### Inclusion criteria

1. Age 9 months to < 21 years
2. Shiga toxin 2 (Stx 2)-positive *E. coli* infection OR a history of bloody diarrhea and evidence of STEC infection OR a presumptive diagnosis of HUS in an individual with a history of diarrhea

#### Exclusion criteria

1. Presence of advanced HUS, defined by:
  - Hematocrit < 30% AND
  - Platelet count < 150 × 10^3^/mm^3^ AND
  - Creatinine > 2.0 mg/dL (177 µmol/L)
    i) The presence of only 1 or 2 of these criteria will not result in patient exclusion, regardless of how close the 3rd criterion is to meeting the exclusion criteria.
2. Prior episode of HUS or diagnosis of atypical HUS [36]
3. Chronic disease limiting fluid volumes administered (e.g., impaired kidney, liver, or cardiac function, chronic lung disease)
4. Evidence of anuria (i.e., no urine output for > 24 h)
5. Hypoxemia requiring oxygen therapy
6. Hypertensive emergency defined as:
  - Elevated blood pressure above the 99th percentile for age [37]
    AND
  - Evidence of neurological change (any of the following)
    i) Seizure
    ii) Diplopia or blurred vision
    iii) Severe vomiting
    iv) Hemiplegia
    OR
  - Severe chest pain with any of the below:
    i) Cardiomegaly on chest X-RAY
    ii) Left ventricular hypertrophy on electrocardiogram or echocardiogram
    iii) Pleural effusions on X-ray

7) Greater than or equal to 10 days since onset of diarrhea or if no diarrhea then the onset of other symptoms [19]
8) Known pregnancy
9) Language barriers impairing appropriate conduct of the study protocol and/or inability to obtain informed consent

### Who will take informed consent? {26a}

#### Additional consent provisions for collection and use of participant data and biological specimens {26b}

**Fig. 2:**
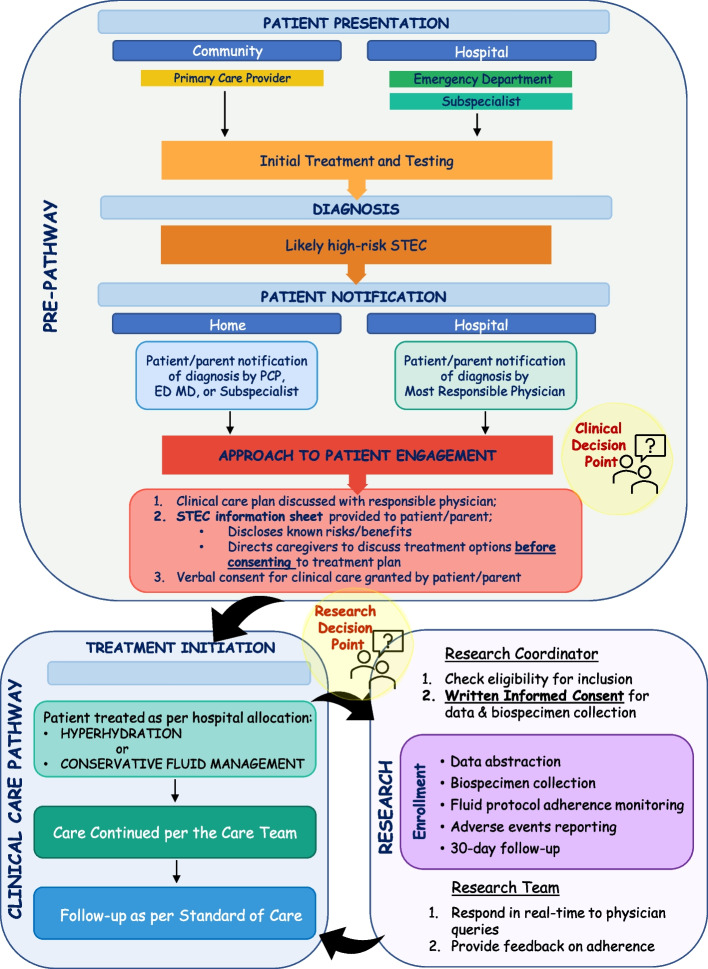
Illustration of the participant flow through the study. Clinical decision point: patients and caregivers are presented with the standard medical risks associated with STEC infection, the known pros/cons of available treatment options, and the treatment currently recommended due to the study allocation. Research decision point: patients and caregivers can consent to data and biospecimen collection

A key tenet of this study is that clinical care is a clinical decision with children receiving care in accordance with the pathway allocated to the site. However, prior to collecting any patient data or biospecimens, informed consent is required along with assent as appropriate. To enable this approach, once a child is identified as being pathway eligible, a member of the clinical care team will provide patients/caregivers with an information sheet (Appendix). This document states that the site is participating in a study and it includes information regarding the institution’s allocated pathway, that there is no accepted standard of care, and the pros and cons of the two treatment pathways. Patients/caregivers will discuss whether to follow the allocated pathway and the alternate options with their responsible physician. Regardless of the clinical decision made, the research team will be alerted to the potentially eligible participant and they will reach out to the responsible physician and request permission to contact the family. If obtained, a member of the research team will approach the family to obtain research consent (Fig. 2) to permit data and biospecimen collection along with consent for the conduct of future studies with the biospecimens and to re-contact the participant for future ancillary studies.

## Interventions

### Explanation for the choice of comparators {6b}

Both treatment pathways reflect widely accepted, albeit usually implemented in a non-standardized manner, treatment approaches used in North American Institutions. As it is unknown which approach is most beneficial, there is equipoise. Both pathways have been reviewed and accepted by critical stakeholders at each institution (including emergency medicine, nephrology, gastroenterology, infectious diseases and hospital medicine) as reasonable and appropriate care options. Embedding protocolized management of either clinical approach (i.e., hyperhydration or conservative fluid management) may lead to improvements over current practice, reducing undesirable outcomes by avoiding dehydration, use of antibiotics or antimotility-agents, and by facilitating close follow up and early recognition of complications.

### Intervention description {11a}

#### Fluid protocol #1—hyperhydration

In this pathway, all eligible children are admitted for the administration of intravenous fluids. To rapidly and safely achieve intravascular volume expansion, based on existing evidence [19, 20, 28], the starting intravenous fluid volume will be 200% of maintenance fluids calculated using the Holliday–Segar formula [38] with the rate adjusted based on clinical parameters and targets.

**Fig. 3:**
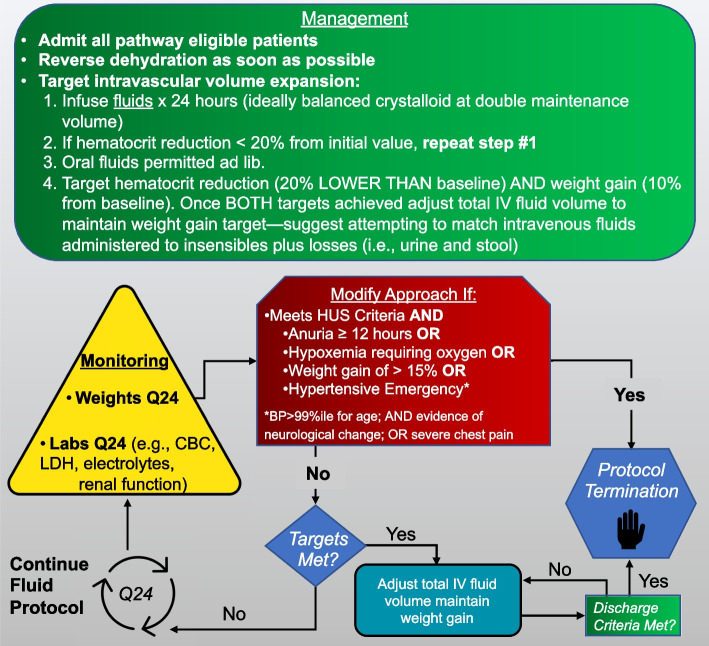
The hyperhydration care pathway recommends the infusion of 200% maintenance fluids until a hematocrit reduction of 20% and weight gain of 10% occur. Patients may be discharged if the following criteria are met even if the targets are not achieved: (1) platelet count is > 50 × 10^9^/L (in absence of transfusion) AND they have increased by ≥ 5%* since the preceding test AND (2) absence of diarrhea (loose or watery stool) × 24 h AND (3) ≥ 5 days since the onset of diarrhea. *If > 10 days since the onset of diarrhea, up to a 5% decrease in platelet count since preceding test is acceptable

The following specifics will form the basis of the fluid management protocol (Fig. 3):

1. Reversal of dehydration: initial rehydration strategies should focus on rapidly reversing dehydration.
2. Infusion of 200% of maintenance fluids × 24 h provided, ideally, as a balanced crystalloid (e.g., PlasmaLyte™, Ringer’s Lactate) intravenous solution. Electrolytes and dextrose may be administered as deemed appropriate; customized solutions are permitted. Intravenous fluid solutions containing < 130 mEq/L sodium should be avoided as they increase the likelihood of developing hyponatremia and are less effective in achieving intravascular volume expansion.
3. If the hematocrit reduction is < 20% from the initial value at 24 h, and the weight gain is < 10%, repeat step #2 (infusion of 200% maintenance intravenous fluids for 24 additional hours) unless the weight gain is ≥ 10% in which case move to step #5.
4. Oral fluids permitted ad lib.
5. If the target hematocrit reduction (20% decrease from baseline) AND a 10% weight gain are achieved, adjust total intravenous fluid volume to maintain targeted weight gain and prevent worsening fluid accumulation (i.e., replace insensible losses plus urine and stool output). If both targets are not achieved, then return to step #2.
  ◦ Insensible losses will be estimated to be 400 mL/m^2^ and these losses should be replaced, along with additional losses through output, every 4 to 6 h [39].
  ◦ Daily weights should be performed and be used to guide the assessment of fluid status with additional fluid adjustments made, as required, to maintain the targeted weight gain.
6. Complete blood count, electrolytes, and kidney function should be repeated every 24 h (or more frequently as deemed clinically indicated).
7. Consider nephrology consultation for all children.

#### Safety targets

Based on the use of this fluid protocol in children with HUS who experienced a mean weight gain of 12.5% without any serious adverse events (SAEs) [18], we have identified parameters ((1) weight gain of 10% AND (2) evidence of hemodilution (i.e., 20% reduction in hematocrit)) which will serve to indicate that intravascular volume expansion has been achieved. This dual parameter approach ensures that hemodilution does not simply reflect hemolysis due to the ongoing microangiopathic process while also identifying a maximal weight gain target to help limit the risk of volume overload.

#### Fluid protocol #2—conservative fluid management

**Fig. 4:**
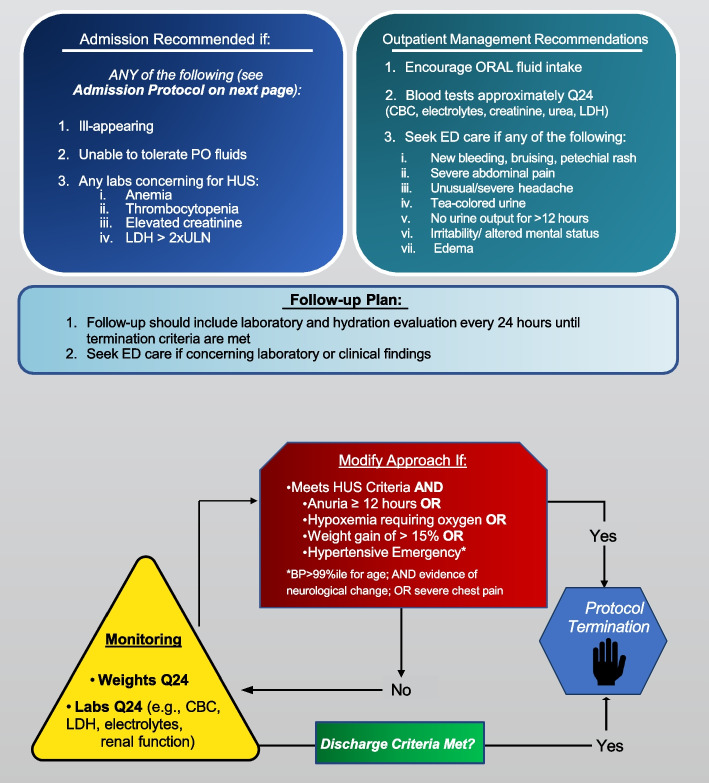
The conservative fluid management pathway leaves the decision to admit the child to the discretion of the clinical care team. If microangiopathy is present admission is encouraged. For inpatients and outpatients, laboratory monitoring should be performed a minimum of every 24 h until discharge criteria are met. If intravenous fluids are administered, the maximum rate is 100% of maintenance and the target is euvolemia and maintaining weight gain below < 5%. The pathway should be discontinued once the same criteria are achieved as for the hyperhydration pathway (Fig. 3)

The conservative fluid protocol has been designed to align and integrate into existing practice patterns. Clinicians can manage children as they normally would with a target of euvolemia. In this pathway, children will undergo a protocolized baseline evaluation that includes reversal of dehydration, if present, and close laboratory monitoring (Fig. 4). The need and approach to reverse dehydration, if present, is at the discretion of the clinical care team. In the absence of evidence of microangiopathy (i.e., normal urinalysis, lactate dehydrogenase, hemoglobin and platelet counts, and creatinine concentrations), the decision to admit or discharge the child is at the discretion of the clinical care team. If microangiopathy is present, admission for monitoring is recommended.

#### The hospitalization protocol includes

1. Reversal of dehydration: initial rehydration strategies should focus on rapidly reversing dehydration.
2. Targeting euvolemia: intravenous fluids should be administered at a maximum rate of 100% maintenance with the rate adjusted to take into consideration ongoing losses (e.g., vomiting, diarrhea, third spacing) and oral fluid intake.
  ◦ The intravenous fluid administered should be isotonic with the volume calculated employing the Holliday–Segar formula [38] with adjustments made as needed to target euvolemia.
  ◦ The fluids provided should be balanced crystalloid (e.g., PlasmaLyte™, Ringer’s lactate) with electrolytes and dextrose added as deemed appropriate; customized solutions are permitted. Intravenous fluid solutions containing < 130 mEq/L sodium should be avoided as they increase the likelihood of developing hyponatremia.
3. Monitoring
  ◦ Daily weights should be performed and be used to guide the assessment of fluid status with additional fluid adjustments made, as required, to maintain euvolemia and weight gain < 5% above baseline.
  ◦ Complete blood count, electrolytes, and kidney function should be repeated every 24 h.
4. Consider nephrology consultation for all children.

#### The outpatient management protocol includes

1. Reversal of dehydration: initial rehydration strategies should focus on rapidly reversing dehydration, if present.
2. Targeting euvolemia: oral fluids permitted ad lib.◦
  ◦ Encourage oral fluid intake as per routine gastroenteritis dehydration management approaches.
3. Monitoring:
  ◦ Complete blood count, electrolytes, and kidney function should be repeated every 24 h.
  ◦ Emergency department evaluation required if laboratory tests or clinical symptoms concerning for HUS. These include any of the following:
    ◾ New bleeding, bruising, petechial rash
    ◾ Severe abdominal pain
    ◾ Unusual/severe headache
    ◾ Tea-colored urine
    ◾ No urine output for 12 h
    ◾ Irritability/ altered mental status
    ◾ Edema
4. Outpatient management otherwise as per the clinical care team.

#### Goal

Once dehydration has been reversed, fluid targets should aim to maintain euvolemia and avoid dehydration.

### Criteria for discontinuing or modifying allocated interventions {11b}

Treatment decisions will be at the discretion of the responsible physician who may choose to deviate from to the allocated pathway. Clinical events and participant preference may lead to care modifications; these would not affect the conduct of study activities (e.g., follow-up). Specific documentation will be made regarding allocated pathway adherence, participant withdrawal, and study discontinuation.

#### End-of-fluid protocol criteria

The pathways will end when the at-risk window for developing HUS, or worsening HUS if present, has passed and this time point is defined by:

1. Platelet count is > 50 × 10^9^/L (in the absence of transfusion) AND
2. Platelets have increased by ≥ 5%* (in the absence of transfusion) since preceding test AND
3. Absence of diarrhea (loose or watery stool) × 24 h AND
4.  ≥ 5 days since the onset of diarrhea

*If >10 days since the onset of diarrhea, up to a 5% decrease in platelet count since preceding test is acceptable.

In addition, the fluid pathways should no longer be followed in children meeting any of the following criteria:

1. Decision to initiate RRT
2. Anuria for ≥ 12 h
3. Hypoxemia requiring supplemental oxygen
4. Weight gain > 15%
5. Hypertensive emergency

If *any of* these criteria are met, the treatment pathway will not resume once they are resolved; care tailored to each patient should be provided as directed by the responsible physician.

### Strategies to improve adherence to interventions {11c}

Site investigators and champions at each site, which include a pediatric emergency medicine physician, nephrologist, pediatric hospital medicine specialist, and at certain sites a gastroenterologist, and/or infectious disease specialist, will be responsible for educating all relevant individuals (at the participating site and in their catchment/referral area) including attending physicians and trainees regarding the treatment pathways. All pathway eligible children will be monitored in real-time, and support will be provided to the clinical care team regarding pathway adherence which has been facilitated through the building and implementation of clinical care order sets embedded into the electronic medical record (EMR) at participating sites.

For each patient, the EMS (emergency medical services) Data Center (EDC, Salt Lake City, UT) will classify the amount of fluid administered in relation to their target as defined by the pathway to which the site has been allocated. Adherence at an individual patient level will be reviewed during weekly leadership team meetings and those cases where participants met our study definition of non-adherence (> 110% and < 175% maintenance fluids in the conservative fluid management and hyperhydration groups, respectively) will undergo review to identify potential etiologies and solutions in an attempt to mitigate future such events.

### Hyperhydration

Data related to all intravenous fluids administered will be extracted from the medical record. For the calculation of pathway adherence, the intravenous fluid administration period begins (i.e., time of study initiation) at the:

1. Time of hospital admission plus 2 h (for participants asked to come to the hospital)* OR
2. Time the participant met eligibility criteria plus 2 h (for participants already in hospital)* OR
3. Actual time of initiation of intravenous fluid administration if occurs before the additional 2-h period has lapsed

*Some participants will be admitted into the hospital before eligibility, and some will become eligible before admission (i.e., positive STEC result in child who is at home). The later of the time points between options (1) and (2) will be used to determine the timing of study initiation if (3) does not apply.

The intravenous fluid administration period ends (i.e., time of study end for pathway adherence determination) when any fluid protocol termination criteria are met (i.e., weight/hematocrit targets, complication ensues, end-of fluid protocol criteria) or after 48 h of fluid administration—whichever happens first. The targeted volume of fluid is defined by the administration of ≥ 175% of maintenance fluids calculated according to the Holliday–Segar formula for the duration of time the participant was in the volume expansion phase of the protocol [38]. Thus, adherence is defined as the administration of fluids in excess of: 100% maintenance fluids × 1.75 × number of hours the participant was in the volume expansion phase of the protocol. Boluses will be included in the quantification of total fluids. Exact monitoring and recording of oral fluid intake are logistically prohibitive and will not be included in the fluid calculation.

The management of patients who receive < 175% of maintenance intravenous fluids in the hyperhydration arm will be classified as nonadherent. However, all patients will be included in the intent-to-treat (ITT) analyses regardless of the volume of fluids administered; secondary analyses will include a per protocol adherence analysis.

### Conservative fluid management

Outpatient/emergency department: All these participants will be considered adherent.

Inpatient: Adherence is defined by the administration of < 110% of maintenance fluids calculated according to the Holliday–Segar formula [38] during the period of time when the participant was in the fluid administration phase of the pathway (defined as per hyperhydration). Thus, adherence to the conservative fluid management pathway is defined as the administration of fluids at or below the following threshold: 100% maintenance fluids × 1.1 × number of hours the participant was in the fluid administration phase. Boluses will be included in the quantification of total fluids. Exact monitoring and recording of oral fluid intake are logistically prohibitive and will not be included in the fluid calculation.

The management of patients who receive > 110% of maintenance intravenous fluids will be classified as nonadherent. However, all patients will be included in ITT analyses regardless of the volume of fluids administered; secondary analyses will include a per protocol adherence analysis.

### Relevant concomitant care permitted or prohibited during the trial {11d}

We will permit the concomitant administration of all medications, supplements, complementary and alternative therapies, treatments, and/or procedures as deemed necessary by the responsible treating physicians. The study will not supply or recommend any specific rescue medication. As part of the overall site education strategies, we will discourage the use of antibiotics, opioids, antimotility agents, and nephrotoxic agents such as non-steroidal anti-inflammatory drugs. We will discourage the use of hypotonic intravenous fluids and will recommend balanced solutions instead of 0.9% saline. However, as this is a pragmatic embedded clinical trial, the treating clinicians, with input from patients and guardians will be responsible to make all treatment decisions.

All relevant data will be extracted daily during chart reviews to enable clarification and confirmation in real-time, if required, and will include concomitant medications administered during the patient’s current illness and any antibiotics taken in the past 14 days. By collecting this information and incorporating it into study analyses, we will be able to identify the independent effects of administered concomitant medications and study interventions.

### Provisions for post-trial care {30}

As study participation involves only data and biospecimen collection, there are no anticipated harms associated with study participation. All participants will receive standard of care as per their hospital’s routine care of children with STEC infection.

### Outcomes {12}

#### Primary outcome: MAKE30 (major adverse kidney events by 30 days)

Because clinical research evaluating the prevention and treatment of AKI has been hampered by the unclear relationship between acute changes in kidney function and longer-term outcomes, the National Institute of Diabetes and Digestive and Kidney Diseases (NIDDK) [40] workgroup on Clinical Trials in AKI recently recommended the use of the MAKE30, a composite endpoint, for phase III trials related to AKI [25]. It is defined by:

1. Death OR
2. Provision of dialysis OR
3. Sustained loss of kidney function (at 30 days) reflected by a 100% [41] increase (i.e., doubling) of serum creatinine from baseline

This outcome will be classified following the completion of data extraction and the 30-day follow-up assessment (time 0, as defined above, is used to calculate the 30-day interval). It will be adjudicated based on laboratory values, chart documentation, and 30-day laboratory results. As baseline creatinine values are rarely available for children, all participants will be assigned a value which will be calculated employing a standardized baseline glomerular filtration rate (GFR = 120 ml/min/1.73m^2^) [42].

#### Outcome justification

The rationale for selection of the MAKE30 as the primary outcome is twofold: (1) the kidneys are the major target organ through which STEC infections cause adverse outcomes and (2) the kidneys are the organ system most likely to benefit from the proposed intervention. Several prior cohort studies demonstrated that RRT use, a component of the MAKE30, is reduced with the use of aggressive intravascular fluid administration in STEC-infected children [18–20]. In addition, the MAKE30 is a widely used and easily assigned composite endpoint of AKI as it includes variables related to kidney prognosis and is based on objective endpoints (i.e., death, provision of RRT, sustained loss of kidney function at 30 days) and thus is unlikely to be influenced by knowledge of study group allocation. The MAKE30 outcome has been used in several recent high profile acute care trials focusing on AKI [41, 43]. In addition, pediatric sepsis survivors who experience the MAKE30 outcome have a three- to fivefold higher rate of developing chronic kidney disease [44].

#### Secondary outcomes

These will focus on quantifying other measures of efficacy and safety. They have been selected to provide supportive information about the effect of volume expansion on the primary outcome and the natural history of disease (see Table 1).

**Table 1:** Outcomes

Outcome measures			
Type	**Name**	**Time frame**	**Brief descriptions**
Primary	Major adverse kidney events to 30 days (MAKE30)	30 days	MAKE30 is defined as: 1) Death due to any cause censored at 30 days after enrollment OR 2) Provision of RRT, any modality, within 30 days of trial enrollment OR 3) Sustained loss of kidney function (100% increase of serum creatinine from baseline at 30 ± 7 days) Baseline serum creatinine: eGFR (120 ml/min/1.73m^2^) will be used to impute an estimated baseline value. This estimate will be applied to all patients as most patients will not have a baseline value from within the 3 months preceding study 30-day serum creatinine: these will only be required for participants with an elevated value at the time of fluid pathway termination who do not receive RRT
Secondary	Significant extrarenal complications of HUS (life-threatening)	30 days	a. Neurologic i. Seizures requiring anticonvulsant therapy ii. Coma: depressed mental state requiring mechanical ventilation for airway protection and/or ventilatory support iii. Thrombotic or hemorrhagic stroke confirmed by neuroimaging b. Cardiac i. Myocardial infarction: elevated troponin and electrocardiographic features of myocardial ischemia with cardiologist assignment of myocardial infarction ii. Myocarditis: elevated troponin along with cardiologist assigned diagnosis of myocarditis iii. Myocardial dysfunction: reduced ejection fraction requiring inotropic agents iv. Cardiopulmonary arrest requiring cardiopulmonary resuscitation and/or extracorporeal membrane oxygenation v. Arrhythmias requiring electrical or chemical cardioversion or pharmacological anti-arrhythmic therapy c. Respiratory i. Respiratory failure requiring invasive mechanical ventilation (i.e., via an endotracheal tube) ii. Pleural effusions requiring thoracostomy or thoracentesis d. Gastrointestinal i. Hyperglycemia requiring insulin therapy at time of hospital discharge ii. Bowel obstruction/perforation requiring surgical repair iii. Intussusception requiring reduction iv. Acute cholecystitis requiring decompression v. Pancreatitis with lipase > 3.0 × upper limit of normal (ULN) for age vi. Hepatitis/liver failure when any one of the following biochemical criteria are met: bilirubin (when other liver functions are in the normal range) > 3.0 × ULN; bilirubin (when accompanied by any increase in other liver function test) > 1.75 × ULN; AST, ALT, and GGT > 8 × ULN vii. Ascites requiring paracentesis a. Infectious complications i. Bacteremia with or without hypotension requiring inotropic support ii. Peritonitis with or without hypotension requiring inotropic support
Secondary	Development of HUS of any severity (i.e., including those who do not meet the MAKE30 endpoint among those without HUS at randomization)	30 days	HUS is defined as [19]: 1) Anemia (hematocrit level < 30%) AND 2) Thrombocytopenia (platelet count < 150 × 10^3^/mm^3^) AND 3) AKI (serum creatinine concentration > upper limit of reference range for age)

### Participant timeline {13} (Table 2)

**Table 2:**
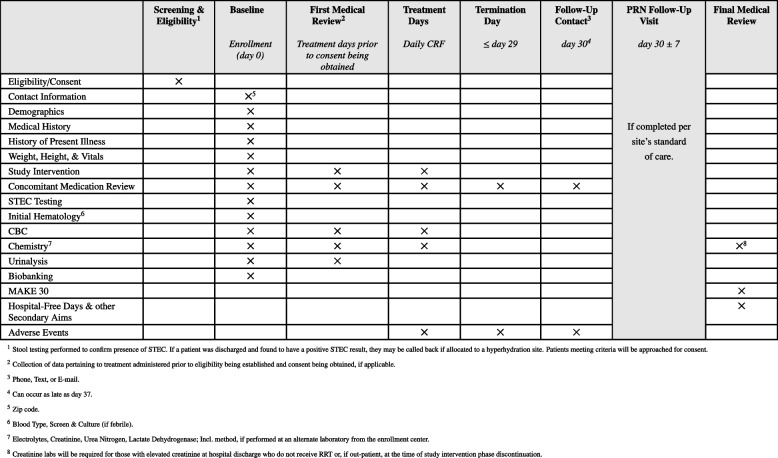
Schedule of activities

^1^Stool testing performed to confirm presence of STEC. If a patient was discharged and found to have a positive STEC result, they may be called back if allocated to a hyperhydration site. Patients meeting criteria will be approached for consent

^2^Collection of data pertaining to treatment administered prior to eligibility being established and consent being obtained, if applicable

^3^Phone, text, or e-mail

^4^Can occur as late as day 37

^5^Zip code

^6^Blood type, screen, & culture (if febrile)

^7^Electrolytes, creatinine, urea nitrogen, lactate dehydrogenase; Incl. method, if performed at an alternate laboratory from the enrollment center

^8^Creatinine labs will be required for those with elevated creatinine at hospital discharge who do not receive RRT or, if out-patient, at the time of study intervention phase discontinuation

### Sample size

#### Target study sample size

We estimate the overall study sample size required is 1040. The number to be screened will likely be 25% higher given that some children will be ineligible, and some will decline consent. Preliminary data indicate that the primary outcome event rate in the conservative fluid management arm will be approximately 10%. The study will be powered to detect a 5% absolute reduction (risk ratio 0.5) in the rate of MAKE30 in the hyperhydration arm. Using a conservative estimate of 5–15 subjects per year per site, we used simulated data (R software) to generate a power table (Table 3) for a 20- or 26-site cluster-crossover design under varying underlying intra-class correlation (ICC) values. The table shows that power is not highly dependent upon ICC and that 20 and 26 sites are required for 80% and 90% power, respectively, at the ICC estimated from preliminary data (i.e., 0.106) [16]. We expect very low levels of missing data, as participants with elevated creatinine values at the time of discharge will have laboratory follow-up as part of their standard clinical care. Patients who deteriorate and develop the MAKE30 outcome will be readily identified. Thus, the impact of loss to follow-up on power is expected to be minimal.

**Table 3:** Power calculations for the primary outcome

Intraclass correlation coefficient	20 cites (approx. 800 subjects)	26 sites (approx. 1040 subjects)
0.05	82%	89%
0.106^a^	81%	90%
0.15	85%	94%

^a^Estimate from preliminary data [16]

The anticipated HUS rate in the conservative fluid management arm is 17.5%, and the rate of extrarenal complications is 7.5%. Detectable absolute differences for these two outcomes with 80% and 90% power, at approximately the same ICC as estimated for the primary outcome, are provided (Table 4). Here, a conservative Bonferroni correction (alpha = 0.025) is used.

**Table 4:** Power calculations for secondary outcomes

Outcome	Power	Detectable difference with 20 sites (*n* = 800)	Detectable difference with 26 sites (*n* = 1040)
Hemolytic uremic syndrome—17.5% in conservative fluid management arm	80%	7.2%	6.3%
	90%	8.1%	7.1%
Extrarenal complications—7.5% in conservative fluid management arm	80%	4.7%	4.1%
	90%	5.2%	4.6%

### Recruitment {15}

#### Source of participants

Research teams will seek to be notified and obtain consent to approach all eligible high-risk, STEC-infected children within their catchment area. Study sites are all leading academic tertiary care centers and leads will disseminate their allocated pathway and a desire to serve as a clinical care site for all STEC-infected children. They will connect with satellite institutions and laboratories to be informed of all potentially eligible children. At their local institutions, through mechanisms put in place with their laboratory and all relevant clinical care teams (e.g., ED, infectious disease, nephrology, gastroenterology, pediatrics), the research team will be notified of potentially eligible children. Study teams will take advantage of their electronic medical records to set up reports that inform them of all positive stool tests and relevant diagnoses (i.e., bloody diarrhea, STEC, HUS). The cluster design promotes the initiation of the allocated treatment at any time and does not require research team involvement of participant research consent. The latter can be obtained following treatment implementation. In addition, consent can be obtained for data collection electronically, and as such, children meeting all eligibility can be enrolled without having to come to the study institution (i.e., they can be treated in accordance with the allocated pathway either at a satellite institution or by a care provider who is willing and able to adhere to the pathway).

#### Recruitment venues

Recruitment into the randomly allocated intervention (i.e., hyperhydration vs. conservative fluid management) will occur in the following venues:

1. Emergency departments
2. Inpatient units: general pediatrics, nephrology, gastroenterology, infectious diseases
3. Medical microbiology laboratories: partnering laboratories will develop a process to provide the research team with the contact information for the ordering physician so that the allocated pathway can be shared to guide care and to request permission to contact the patient/caregivers of children with positive STEC specimens to obtain research consent. This approach will enable participating sites to recruit participants from their broader geographic catchment area.

#### How potential participants will be identified and approached

**Fig. 5:**
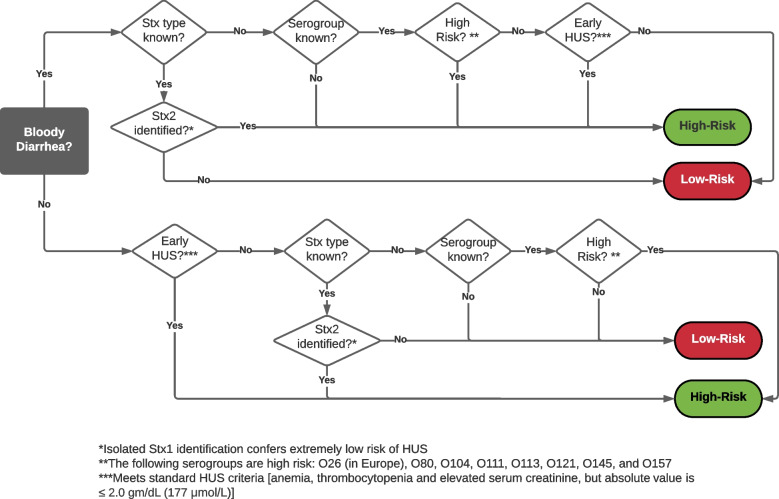
Approach to optimize the enrollment of high-risk STEC infected participants

Participant identification/approach will vary between sites, but will be based on the following key concepts:

1. Clinician identification: once a child is identified as having a high-risk STEC infection, clinicians will provide patients/caregivers with an STEC information sheet which clearly states that the site is participating in this study, the institution’s treatment approach within the study, and the pros/cons of the two treatment protocols. Clinicians at all sites will be trained to contact the appropriate research team member to determine research eligibility and to obtain permission for the research team to approach the caregiver to request informed consent for data extraction and biospecimen collection.
2. Research team identification:
  A) Daily screening: reports from the ED and inpatient units will be created to identify all children with potentially eligible chief complaints (e.g., bloody diarrhea, abnormal laboratory result) and discharge diagnoses (e.g., STEC, HUS). Patients infected with a high-risk STEC (evidence of an *E. coli* O157:H7, detection of Stx 2 or a gene encoding this toxin, bloody diarrhea, and presence of Stx or a Shiga toxin gene not otherwise specified, or evidence of early HUS) will be identified (Fig. 5). The research team will approach the clinical team caring for such patients to review research eligibility and to obtain permission to approach the patient for research consent.
3. Medical microbiology laboratory identification: reports identifying all STEC positive stool specimens will be set up. For all children identified potentially eligible children, contact will be initiated by the research team with the responsible physician who will be informed of the sites allocated pathway and who will be asked to obtain consent for the research team to approach the family.

For all discharged and outpatients, the child will be asked to go to the ED (if required for the hyperhydration arm) for screening (i.e., baseline laboratory testing) and treatment per the institution allocated pathway. If in the conservative fluid management arm, at the discretion of the responsible physician, the child may be managed as an outpatient and consented for research electronically.

#### Targeted outreach activities

To optimize awareness of the embedded pathways at participating sites to optimize both pathway adherence and study awareness to promote recruitment, we will conduct knowledge dissemination activities aimed at the following groups:

1. Physicians (particularly pediatricians, hospitalists, emergency physicians, nephrologists, gastroenterologists, infectious disease) and nurses at the participating center. These strategies will include emails, lectures, seminars, section/department meetings, creation of order sets, and clinical care guidelines.
2. Physicians providing primary care, hospital-based care, and emergency department care in the institution catchment area—through emails, lectures, seminars, section/department meetings, and the sharing of order sets and clinical care guidelines.
3. Medical microbiology laboratories—through direct communication with laboratory directors to optimize diagnostic testing approaches and mechanisms to become notified of positive test results.

## Assignment of interventions: allocation

### Sequence generation {16a}

The EDC will generate the randomized assignment sequence for the hospitals. A geographically stratified approach will be used with sites grouped into six zones based on country and geographic proximity (Fig. 1), with half the hospitals in each zone assigned to each arm. Participating centers will implement the first assigned intervention until a total study target number of patients is reached. Following a simultaneous crossover, in the second phase, centers will implement the second intervention in a similar number of consecutive patients. The crossover point and timing is planned to occur after 24 months of recruitment with the precise date being determined by the EDC based on actual recruitment.

### Concealment mechanism {16b}

Study site allocation was delayed as long as possible to ensure that sites had signed agreements to participate and final study sites had been identified, ethics approval obtained, and study launch confirmed. Site allocation was revealed on May 6, 2022.

### Implementation {16c}

The allocation sequence was generated by the study’s lead biostatistician Dr. Casper. Participants will be started on the care pathway by the responsible health care providers. Participants will be enrolled by research team members. As it is a cluster randomized trial, no allocation will occur at the patient level as it is pre-determined for the site.

## Assignment of interventions: blinding

### Who will be blinded {17a}

This will be an unblinded study. Assessment of the primary endpoint, MAKE30, is objective and unlikely to be influenced by lack of blinding. Participating sites will be provided with their allocated pathway that will be embedded into clinical care, thus blinding is not feasible.

### Procedure for unblinding if needed {17b}

This is not applicable as this is an unblinded study.

## Data collection and management

### Plans for assessment and collection of outcomes {18a}

Plan for assessment of study outcomes is summarized in Table 1 and Sect. 12 (Outcomes).

### Plans to promote participant retention and complete follow-up {18b}

Baseline symptom data will be collected from participants; testing, treatment, and outcome data will be extracted from the medical record. A 30-day (± 7 days) evaluation of renal function is required in the small subset of participants who have an elevated creatinine at the time of pathway termination in children who do not require RRT, to determine the MAKE30 primary outcome. This laboratory evaluation is considered standard of care and thus, no specific research follow-up is required.

A participant will be considered lost to follow-up if a 30-day follow-up serum creatinine value is required to assign MAKE30 status, and none is performed as part of clinical care, or the result cannot be obtained. To minimize lost to follow-up, the following actions will be taken by the clinical care team if a participant fails to return to the clinic for the required follow-up:

1. The clinic will attempt to contact the participant. If contact is made, the clinical care team will counsel the participant on the importance of serum creatinine monitoring. If the participant is unable or unwilling to attend the study site phlebotomy laboratory as directed, the site will provide an alternate, more convenient location for serum creatinine testing.
2. If step 1 is unsuccessful, the research team will make every effort to regain contact with the participant, employing, where possible, up to 3 telephone calls and, if necessary, a certified letter to the participant’s last known mailing address or local equivalent methods. This contact will serve to reinforce the importance of clinical follow-up and we will re-connect the participant with the clinical care team.

Non-adherence with the allocated fluid administration protocol by the clinical care team will not impact research data collection and study procedures; we will collect all primary and secondary outcomes data as well as AEs, SAEs, and unanticipated problems (UP) regardless of adherence status. Participants are free to withdraw from the study at any time thereby terminating further data collection. The reason for participant discontinuation or withdrawal will be recorded.

### Data management

Data collection is the responsibility of research team members at participating sites under the supervision of the local site investigator who is responsible for ensuring the accuracy, completeness, legibility, and timeliness of the data reported. The EDC has created paper and electronic data collection forms for utilization and a detailed Manual of Operations (MOO) that describes source documents, instructions for completing the case report forms, and relevant data handing and monitoring procedures.

Research data will be entered into the study’s REDCap database which is password protected and includes internal quality checks built to identify data that are inconsistent, incomplete, or inaccurate. A risk-based monitoring plan details the data monitoring strategy and procedures. All statistical analyses will be coded and conducted independently by two statisticians.

### Confidentiality {27}

Participant confidentiality and privacy is strictly held in trust by the participating investigators, their staff, and the EDC. No information concerning the study or the data will be released to any unauthorized third party without prior written approval of the sponsor. All data entered into the REDCap database will be encrypted and transmitted to the EDC, linked to a unique research identification number, where it will be stored. No identifiable participant information will be transmitted. All biospecimen samples will be linked to research data by a unique research identification number. Personal health information (PHI) will not be shared with any third parties. Research records at local sites will be locked file in a secure room unless being used by research staff, and all computerized information will be maintained on password-protected computers and archived on a secure server. Security measures will be dictated by local regulatory requirements, including but not limited to locked cabinets in locked offices, and 128-bit or greater encryption on secure computers. Only site investigators, research coordinators, and qualified key personnel will have access to these records. Electronic transmission of de-identified records over public networks will be encrypted with virtual point-to-point sessions using SSL or VPN technology. Data will be analyzed and published in aggregated and encrypted form only.

### Plans for collection, laboratory evaluation, and storage of biological specimens for genetic or molecular analysis in this trial/future use {33}

With informed consent, and as approved by local IRBs/REBs, de-identified biological samples will be stored to be used to explore the pathophysiology of HUS in STEC-infected patients and its complications. During, and after the conduct of the study, an individual participant can choose to withdraw consent to have biological specimens stored for future research. A code-link will be created to link biological specimens with clinical data, thereby enabling analyses while maintaining blinding to participant identity. Following study completion, access to study data and/or biospecimens will be determined by the study’s leadership team. IRB/REB approval will be required for all studies involving biospecimens. Genetic testing of blood samples will not be performed.

## Statistical methods

### Statistical methods for primary and secondary outcomes {20a}

Baseline characteristics of subjects will be summarized by intervention group. Counts and proportions will be used for categorical variables and means, standard deviations, medians, and quartiles will be used for numeric variables.

We will adhere to the ITT principle. The primary outcome, the MAKE30, will be compared between children allocated to hyperhydration vs. those allocated to conservative fluid management. We will use logistic regression with generalized estimating equation (GEE) methods to account for clustering at the site level. Fixed model terms will include treatment effect and period effect. Other baseline terms in the model will be age (as a continuous variable), serum creatinine, and hematocrit. These variables have been associated with adverse kidney outcomes in this population. The superiority test of null treatment effect will be carried out at the 0.05 level. Parameter estimates and 95% confidence intervals will be provided for all model terms. Carry-over effect will not be present in our crossover design in the traditional sense as patients themselves, and therefore, their outcomes will only be subject to one type of treatment. The only possible “carryover” effect would be a lack of adherence to the new treatment protocol after the crossover at the site level. We will monitor this closely and its potential effect which would be to reduce power.

The primary outcome, MAKE30, is a composite measure that includes (1) death, (2) new initiation of RRT, and (3) persistent kidney dysfunction. The latter is traditionally defined by a persistent increase in creatinine from a known baseline value at 30-day follow-up. However, most children with STEC-HUS are previously healthy and do not have baseline serum creatinine values available. As such, we will compare 30-day values to estimated, pre-illness values. Although imputation methods have been used previously in critically ill children to estimate baseline serum creatinine values, most of these require patient height. However, as children managed as outpatients under the conservative fluid management strategy will not routinely have height documented, this approach also would be a challenge to our study. Pottel et al. [45], however, established and validated a height-independent equation that performs similarly to height-dependent equations as a screening tool for kidney dysfunction [46, 47]. We will use this approach to calculate estimated creatinine values and will define persistent kidney dysfunction as a 50% increase over these estimated values.

Secondary outcomes will be analyzed in a similar manner as the primary outcome, with the dichotomous outcomes of extrarenal complications and HUS utilizing the same logistic regression model. Only patients without HUS at baseline will be included in the latter analysis. Additional details can be found in the HIKO STEC Statistical Analysis Plan.

### Interim analyses {21b}

The study’s Data and Safety Monitoring Board (DSMB), whose purpose is to protect the safety of participants and promote the scientific integrity of the study, will include five members. The DSMB will monitor study participant accrual, protocol adherence, data quality, site performance, SAEs and other subject safety issues and will review formal interim statistical analyses of treatment efficacy. The EDC will send reports relating to DSMB members prior to each meeting. The EDC will attend DSMB meetings; each meeting will have summary recommendations that will be provided to the principal investigators who will be responsible for responding to all recommendations. The EDC will distribute the final signed recommendation within 3 business days to the investigators, DSMB members, and the federal funding agency and will post the final signed recommendations to the EDC Document Library.

The DSMB had its inaugural pre-launch meeting on June 17, 2022, and an additional meeting was convened on July 29, 2022, to address outstanding concerns prior to study launch. The DSMB will hold two planned meetings to review formal interim analyses to examine treatment efficacy and patient safety. The first formal efficacy analysis will occur prior to the intervention switch (i.e., crossover). This analysis will test the primary hypothesis by using the model described for the analysis of the primary outcome but without a period effect term. In the second intervention phase, a formal interim efficacy analysis will occur after the seasonal peak of STEC incidence. Early stopping of the trial will be considered if efficacy has been established. One-sided Haybittle–Peto boundaries will be used for efficacy monitoring. The trial may be stopped for harm and such analyses will be performed as part of our interim analysis. The DSMB will review accumulating AE data and weigh the potential risks and benefits at each meeting. If there is a perceived increase in risk of AEs with little indication of benefit, the DSMB has the ability to recommend stopping the trial. In addition, they will meet every 12 months to review the protocol, study procedures, monitoring, participant safety, enrollment, and loss to follow-up.

This study may be suspended or prematurely terminated if there is sufficient reasonable cause at an ad hoc DSMB meeting. The following events will trigger a notification to the DSMB chair to determine whether an ad hoc DSMB meeting is warranted to review AEs and to make recommendations to the principal investigators:

1. Mortality frequency > 1% of participants
2. RRT frequency > 15% of participants
3. Intubation frequency > 5% of participants (minimum duration > 24 h)

When considering such AE data, the DSMB will be encouraged to consider the number of participants enrolled to date.

Written notification, documenting the reason for study suspension or termination, will be provided by the EDC to investigators, funding agencies, the central IRB, and local REBs. The notification will include the reason(s) for the termination or suspension. The study may resume once the identified concerns are addressed to the satisfaction of the sponsor, IRB/REBs, and the DSMB.

### Methods for additional analyses (e.g., subgroup analyses) {20b}

For additional insight, we will perform a “confirmatory” analysis restricted to patients with high-risk STEC, defined as meeting one of the following criteria:

1. Evidence of Stx2 infection
2. Evidence of O157, O103, O104, O111, O113, O121, or O145 infection
3. Bloody diarrhea and no toxin or typing available
4. Early HUS and no toxin or typing available

This is the population that would be “ideal” for the trial. However, the identification of this population prior to initiation of treatment is not feasible with current technology and workflow.

The primary outcome will be analyzed across predefined subgroups to look for a differential treatment effect. Subgroups will be based on age (0 to < 5.0, 5.0 to < 10.0, and ≥ 10.0 years), sex, duration of diarrhea prior to pathway initiation (< 96 and ≥ 96 h), and baseline hematocrit (< 40% and ≥ 40%). A subgroup effect will be considered significant if the interaction between subgroup and treatment (added to the same model as used for the primary analysis) is significant at a Bonferroni-corrected level. Exploratory outcomes will be analyzed in the same way as the primary and secondary outcomes with no adjustment for multiple comparisons. When these results are reported, they will be identified as exploratory.

Safety analyses: frequencies of SAEs will be compared between subjects allocated to hyperhydration vs. those allocated to conservative fluid management. The occurrence of any SAE will be considered as a dichotomous outcome. The start date, stop date, severity, relationship, expectedness, outcome, and duration of SAEs will be reported and treatment-associated SAEs will be presented in a table or listing.

### Methods in analysis to handle protocol non-adherence and any statistical methods to handle missing data {20c}

The primary analysis will be performed using the ITT population, consisting of all participants who are enrolled, included within the study arm corresponding to the treatment assigned to the site during that study period. The ITT population will also be the population used in all secondary outcome, safety, and subgroup analyses. A per-protocol analysis will be conducted to gain additional insight, but the results will not replace the results of the ITT analysis. The per-protocol analysis dataset will consist of the subset of the ITT population who are confirmed eligible and are classified as protocol “adherent.”

Per the ITT principle, subjects who withdraw from the study or are lost to follow-up will have all available data used in the analysis. Subjects who are missing data for a particular outcome will not be included in analysis of that outcome. If a substantial number of subjects are withdrawn or lost to follow-up, baseline characteristics and available information on hospital course will be reviewed and compared to subjects not withdrawn or lost, to assess empirically if these subjects differ from those remaining in the study for the scheduled treatment and follow-up time. However, we expect very little missing data for the primary outcome.

### Plans to give access to the full protocol, participant level-data, and statistical code {31c}

The study’s protocol will be published in a peer-reviewed journal, and it will be submitted, alongside the manuscript reporting the main results of the trial, following trial completion. The study will also comply with the NIH’s Data Management and Sharing Policy. In addition, this trial has been registered at ClinicalTrials.gov (NCT05219110), and the record will be updated in accordance with their policies. The statistical code used to analyze study data will be shared upon reasonable request. The data and supporting information will be made available and will remain open access in the public domain within 3 years following completion of the study protocol by the last enrolled participant.

## Oversight and monitoring

### Composition of the coordinating center and trial steering committee {5d}

The organization of the trial leverages the infrastructure, expertise, and leadership of the Pediatric Emergency Care Applied Research Network (PECARN) [48] and Pediatric Emergency Research Canada (PERC) [49] research networks. The organizations have reviewed and provided extensive feedback that has been integrated into the final protocol which they have approved. The PECARN EDC located at the University of Utah will serve as the study’s data coordinating center and will serve as the central organizing site and is responsible for the preparation and maintenance of study training documents. The EDC will liaise with the Central IRB and the DSMB and will conduct all statistical analyses. The EDC is a unit that operates independently from all participating study sites.

The Executive Committee, composed of the study co-PIs (SF, DS, PT), the PI of the EDC (TCC), and co-investigators (SG, SG, AP), will serve as the overall governing body and will be tasked with assuring that major milestones, timelines, and enrollment targets are met. The Executive Committee will work closely with the NIAID Program Officer, EDC, and DSMB in the oversight of the trial.

A separate Steering Committee, composed of the Executive Committee members and the leaders of the PECARN, PERC, and the PEMCRC networks and a pediatric bioethicist (BW), will meet every 6 months. This committee will provide high-level oversight and guidance regarding ethical and logistical considerations, study conduct, and manuscript preparation.

### Composition of the data monitoring committee, its role, and reporting structure {21a}

Safety oversight will be under the direction of the DSMB which is composed of individuals with expertise in pediatric emergency medicine, pediatric intensive care, pediatric nephrology, and biostatistics. Members of the DSMB are independent from study conduct and free of conflict of interest. The DMSB is operating under the rules of an approved charter that was approved at the initial DSMB meeting. Each data element that the DSMB requires to perform its duties has been clearly defined and these will be provided to them by the EDC. The DSMB has provided its input to the study PIs, sponsor, and other regulatory agencies, as appropriate. The funder, represented by an NIAID program officer and medical monitor will be invited to attend open sessions of the DSMB meetings as a consultative non-voting member, and they will receive copies of the minutes and the charter.

### Adverse event reporting and harms {22}

Study research teams will monitor for the occurrence of AEs throughout the study period. AEs may be reported by the subject or caregivers, discovered by investigator questioning, or detected by the clinical care team through physical examination, laboratory test, or other means. The study period is defined as the initiation of treatment (Day 0) through Day 30 (± 7 days). AEs will be recorded on the AE case report form. The nature of each experience, date and time of onset, course, outcome, severity, and relationship to treatment will be established.

Summaries of event rates, intensity, and relationship to study allocation of individual SAEs will be presented by System Organ Class and Preferred Term (MedDRA). MedDRA is a multilingual standardized international medical terminology dictionary used for regulatory communication and evaluation of data pertaining to medicinal products for human use. AE severity will be assessed by the site investigator (Table 5).

**Table 5:** Adverse events severity scale

Severity		
Grade 1	Mild	Transient or mild discomfort (< 48 h); no medical intervention/therapy required
Grade 2	Moderate	Mild to moderate limitation in activity—some assistance may be needed; no or minimal medical intervention/therapy required
Grade 3	Severe	Marked limitation in activity, some assistance usually required; medical intervention/therapy required, hospitalizations possible
Grade 4	Life-threatening	Extreme limitation in activity, significant assistance required; significant medical intervention/therapy required, hospitalization or hospice care probable
Grade 5	Death	

The suspected relationship between study interventions and any AE will be determined by the site investigator. All AEs, including SAEs, will be evaluated as to whether their occurrence was expected or unexpected. An AE will be considered unexpected if the nature, severity, or frequency of the event is not consistent with the known risks associated with the study intervention or with the underlying medical condition (i.e., STEC infection).

The following events and symptoms are routinely expected to occur as part of the natural history of STEC infection, and as such, we a priori determined that they will not be reported as AEs: vomiting, fever, diarrhea, abdominal pain, dehydration, anemia, thrombocytopenia, hospitalization for intravenous rehydration or ongoing oral rehydration therapy, renal insufficiency, HUS, and/or RRT.

### Frequency and plans for auditing trial conduct {23}

Each participating site will perform internal quality management of study conduct, data and biospecimen collection, documentation, and case report form completion. An individualized quality management plan and report will be developed to describe a site’s performance. As per standard practices, prior to the start of participant enrollment, site research staff will receive extensive training in the study’s protocol. Quality control and reliability of screening, baseline data collection, and follow-up will be monitored by the EDC. The results will be shared with the study Executive Committee, and site research coordinators and investigators, via monthly study teleconferences.

Quality assurance will be accomplished in several ways under supervision of the EDC.

- Enrollment metrics: site research team members will enter screening data into the study database. This will enable the EDC to determine the number of patients who qualified for enrollment, the number who met exclusion criteria, and the number who declined to participate in the study.
- Data quality: completeness of participant data will be monitored by the EDC utilizing a real-time data query system, which identifies data deficiencies daily. Sites will receive daily reports on the number of outstanding data queries.
- Retention metrics: participant retention data will be analyzed, and the EDC will issue monthly site-specific reports on participant follow-up success rates.
- Adherence to IRB/REB requirements: participant IDs will randomly be selected, and regulatory documents will be reviewed on a regular basis by the EDC. Specific details are described in the risk-based monitoring plan.
- Accuracy of collected data: assessment of entered data accuracy will be performed during site audits.
- Site monitoring: a risk-based monitoring plan will be followed to ensure overall data quality, maximize detection of non-compliance and/or quality issues, and verify adequate investigator oversight. Findings will be assessed for severity, impact, prevalence, and actions taken by the site to address any concerns identified. The site monitor will communicate findings with each site to ensure understanding and compliance of the protocol and address concerns in a timely and appropriate manner.

If quality assurance issues are detected, the site PIs, project managers, and EDC staff will work collaboratively to address these issues. Participating sites will provide direct access to all trial source data/documents and reports for the purpose of monitoring and auditing by the sponsor and inspection by local and regulatory authorities, as required.

### Plans for communicating important protocol amendments to relevant parties (e.g., trial participants, ethical committees) {25}

Protocol amendments will be communicated to investigators through monthly newsletters and monthly all-site team meetings. These amendments will be submitted to the IRB/REBs by the appropriate site investigators and to the central IRB by the EDC. Study participants will not be informed of study amendments given the brief period of study participation. The trials registration at www.clinicaltrials.gov will be amended by the study team. If indicated, the principal investigator (SF) will inform journals of study amendments. As this study is not regulated by any federal agencies, no regulatory bodies will be notified of amendments.

### Dissemination plans {31a}

We plan to disseminate our results via open access publication in peer reviewed publications and subsequently via traditional (e.g., research conferences) and social media means. The www.clinicaltrials.gov database will be also updated with study results.

## Discussion

The HIKO STEC trial is an embedded, cluster-randomized, crossover trial that will enroll 1040 STEC-infected children to test the hypothesis that hyperhydration is nephroprotective, improves outcomes, and reduces complications when compared to traditional conservative fluid management. This trial is important because HUS develops in up to 20% of children with high-risk STEC infections children and 60% of these children will require RRT. Thus, although STEC infection is one of the most common causes of acquired kidney injury in young children [5, 16, 33], no specific treatments have emerged in the four decades since STEC were determined to cause HUS.

This trial is required because equipoise remains regarding use of hyperhydration in STEC-infected children, particularly in the pre/early-HUS time periods. A cluster-randomized crossover design avoids the logistical issues associated with patient-level randomization given our anticipated timelines, approach to participant identification and recruitment, the multitude of physicians and clinical services involved, and the many locations (e.g., home, emergency department, inpatient) involved in patient care. Thus, after extensive consultation, and in consideration of recommendations provided by grant peer-reviewers, it was concluded that alternating between treatment options at an individual patient level within an institution would be too disruptive and confusing. By implementing a cluster-crossover trial design, the intervention is bundled into clinical care, and families can be at home when the conservative fluid management protocol is initiated. This approach minimizes the burden on families which would be significant in an individual patient-level randomized trial, as treatment would be unpredictable and would require all children to be recruited and consented for care at any time of the day. It would also require that they travel to the study site without knowledge of their treatment plan (e.g., outpatient or inpatient) until their child is randomized. This embedded, pragmatic approach, with research consent required for data and biospecimen collection, is similar to and builds on other NIH-funded trials such as TiME [50], ABATE [51], SMART [41], and SALT-ED [43]. Cluster randomization without crossover and stepped-wedge designs were also considered, but these formats require a greater number of sites to achieve comparable power, which increases the study budget and requires the participation of sites with fewer potentially eligible participants.

Our design is pragmatic; by embedding the allocated treatment pathway into clinical care [41, 43, 50–53], the intervention can commence as soon as clinically indicated to maximize the potential therapeutic benefit. This approach was selected and developed in consultation with the NIH Collaboratory’s Ethics and Regulatory Working Group and approved by our central IRB at the University of Utah and all local IRB/REBs. The clinical care teams at all study sites have agreed to adopt the allocated protocols as their standard care and to crossover to the alternate pathway when instructed to do so. Nonetheless, as with all clinical care decisions, the guardians, and children, as appropriate, will be informed of the study and empowered to be involved in all care decisions. Research consent for data extraction and biospecimen collection will be sought as early as possible.

Participating sites (Fig. 1) were selected with the support of the National Center for Advancing Translational Sciences (NCATS) based on their known number of annual cases of STEC infection and HUS. They include 22 US and 4 Canadian sites that possess the clinical research infrastructure and expertise to successfully participate in this trial. Sites have participated in webinars, identified local investigators, champions, and subspecialty partners, and established collaborative relationships with laboratories and outpatient providers. Selected sites provide broad geographic representation, and they serve racially and economically diverse populations. In addition to serving as childhood AKI referral centers, many have participated previously in multicenter ED-based studies led by the PERC and/or PECARN networks [16, 49, 54–61].

To minimize bias, we will adhere to the guidelines on the conduct of cluster randomized crossover trials [62] and the CONSORT2010 extension to stepped wedge cluster randomized trials [63]. To control for the potential bias introduced by period effects, we will randomize the order in which the interventions are delivered to each cluster. Selection bias will be minimized by ensuring equal periods of recruitment into each arm, and the inclusion of many sites with broad geographic distribution. Participation will not be influenced by local teams as a complete cluster enumeration approach will be employed (i.e., the intervention will be embedded into clinical care and all STEC-infected children will be managed in accordance with the allocated protocol; consent will be obtained for data and biospecimen collection only). Cluster adherence will be monitored, and poor adherence addressed. ITT and as-treated analyses will be performed. Clusters will be unaware of the precise crossover date, which will depend on recruitment; however, we intend for it to occur during the winter, a season with few STEC cases, to minimize treatment protocol contamination. We do not anticipate that this will be a significant problem as individual physicians will develop minimal experience-based bias as each physician will only treat a small number of STEC-infected children as sites will average 10 participants/year total. Lastly, outcome reporting bias will be minimized by publishing the trial protocol and registering it at clinicaltrials.gov.

We have designed this study cognizant of the difficulties inherent to trials of fluid treatment strategies. The categorical assignment of such interventions is complicated by clinical realities including variable and at times severe clinical courses, interval since illness onset, and physician awareness and preferences as it relates to intravenous fluid administration in children with evolving AKI. These factors may generate a range of fluid administration in both arms and the potential for intervention overlap. Nonetheless, the volumes we propose were chosen to encompass the continuum of fluid administered to STEC-infected children in multiple continents (e.g., North America [19, 20], Europe [18, 35], and South America [64]). Our analysis plan incorporates methods to relate outcomes to the allocated treatment arm (ITT analysis), to the volume received (per-protocol analysis), and to the intervention as a continuous variable, regardless of treatment allocation (as-treated analysis). Thus, we will be able to identify the optimal fluid regimen strategy in children with high-risk STEC infections.

## Trial status

Protocol version 3.6 02-06-2023. Recruitment commenced on September 19, 2022, and should be completed by September 2026.

## Data Availability

Although no contractual agreements exist limiting data access for the investigators, the final trial dataset will only be accessible to designated individuals based at the EDC.

## Appendix

### Shiga Toxin- Producing *Escherichia coli*

- Shiga-toxin producing *E. Coli* (STEC) are bacteria (germs) that cause gut infections. Most commonly, STEC infections begin with diarrhea that becomes bloody. These infections can be painful. The bacteria produce a toxin that causes up to 20% of infected children to develop a serious complication called the hemolytic uremic syndrome (HUS). HUS leads to kidney failure in about 60% of children who develop this complication. It can also affect many other organs. In rare cases (1 to 3%), HUS can cause death.
- We do not know the best way to prevent HUS or to lessen damage to children’s kidneys. At this moment, your child does not have kidney failure or require dialysis. Two approaches are used to manage children at this stage. Many physicians recommend that children be monitored at home (and sometimes in hospital if children have severe symptoms) and wait for HUS to occur (or not as is the case for 80% of children), because there is no specific treatment that stops its progression. Some doctors recommend early hospitalization, even in well-appearing children, and administer a large volume of intravenous fluids based on the thought that this can prevent or lessen the severity of HUS.
- We are one of 26 hospitals participating in an international research study sponsored by the National Institutes of Health (NIH) seeking to compare the following two approaches to treating STEC-infections.
  1. Traditional Approach: Children are treated either at home or in hospital based on the severity of symptoms and access to follow-up care and monitoring. The focus is on close monitoring and providing supportive care for HUS complications as early as possible.
  2. Hyperhydration Approach: Children are treated in the hospital, even if they are feeling well. They receive lots of intravenous fluids to maximize kidney blood flow.

Your child needs clinical care, but it is unknown which approach is best. Both approaches are currently in use, each has potential benefits and downsides, and no research has compared the two approaches.

- Our hospital has been assigned to the HYPERHYDRATION approach. We currently admit all STEC-infected children to the hospital, provide intravenous fluids, and closely monitor children until the doctors are confident that HUS is not going to develop. This usually requires 2 nights in hospital. If HUS does develop your child’s treatment will be determined and adjusted directly by their doctor. We hope this approach will prevent the development of HUS or lessen its severity should it occur.Please discuss any questions or concerns you may have with your child’s doctor before agreeing to the proposed treatment.

****Alternative language****

- Our hospital has been assigned to the TRADITIONAL approach. This approach promotes individualized decision making by your child’s doctor who will recommend if your child should be hospitalized or can be followed and managed at home. We believe this approach limits unnecessary hospitalizations, costs and complications from fluid administration. Please discuss any questions or concerns you may have with your child’s doctor before agreeing to the proposed treatment.
- As with usual clinical care, the costs of treatment will be billed to insurance or is the responsibility of the patient. Both approaches reflect accepted clinical practice and the recommended monitoring and treatment are considered routine costs.

## Frequently Asked Questions

### HOW DID MY CHILD BECOME INFECTED?

Shiga-toxin producing *E. coli* (STEC) can be acquired by many different routes:

- Eating contaminated meat or produce
- Swimming in pools or lakes contaminated with feces
- Having close contact with an infected person

### WHAT ARE THE SYMPTOMS OF STEC INFECTIONS?

Most children with STEC will have bloody diarrhea accompanied by abdominal pain, cramping, and bloating. STEC infection may also cause vomiting and fever.

### WHAT CAN HAPPEN TO CHILDREN INFECTED WITH STEC?

HUS is the most common and serious complication of STEC infection. HUS may cause serious kidney damage, and children with HUS might require dialysis. In rare instances, HUS can cause life-threatening complications such as stroke or seizures, clotting problems, heart problems and problems with the intestines, gallbladder or pancreas. If dialysis is started, it is usually temporary (1–2 weeks).

### WHAT ARE THE POTENTIAL BENEFITS OF HYPERHYDRATION?

Some doctors think that giving more fluids early in the disease will prevent or minimize HUS and other complications. This can lessen kidney injury and the need for dialysis.

### WHAT ARE THE POTENTIAL SIDE EFFECTS OF HYPERHYDRATION?

If your child develops kidney failure, they may experience fluid overload which might have unknown complications in children with STEC infection. Your child’s physician will monitor for signs of excessive fluid and will stop the treatment if your child shows any concerning symptoms, such as trouble breathing, excessive weight gain, and swelling. There is also a risk of acquiring a hospital-related infection. There may be risks that are not yet known, but all children will be monitored closely.

### WHAT ARE THE POTENTIAL BENEFITS OF TRADITIONAL FLUID MANAGEMENT?

Most of the time the traditional approach can be conducted at home thereby reducing costs and the risk of acquiring a hospital-related infection.

### WHAT ARE THE POTENTIAL SIDE EFFECTS OF TRADITIONAL FLUID MANAGEMENT?

Dehydration can develop due to inadequate drinking or if they suffer large amounts of vomiting and/or diarrhea. Dehydration is associated with an increased risk of kidney failure in children with STEC infection. All STEC-infected children should have blood tests done every 24 h – this can be more challenging to organize and monitor when children are monitored outside of the hospital. There may be risks that are not yet known, but all children will be monitored closely.

## Abbreviations

AE: Adverse event
AKI: Acute kidney injury
CBC: Complete blood count
CFR: Code of Federal Regulations
CMP: Clinical Monitoring Plan
CONSORT: Consolidated Standards of Reporting Trials
CRF: Case report form
EDC: EMS Data Center
DSMB: Data Safety Monitoring Board
ED: Emergency department
EMR: Electronic medical record
eCRF: Electronic case report forms
FDA: Food and Drug Administration
GCP: Good clinical practice
GEE: Generalized estimating equation
GFR: Glomerular filtration rate
HUS: Hemolytic uremic syndrome
ICC: Intra-class correlation
IRB: Institutional Review Board
ITT: Intention-to-treat
IV: Intravenous
MAKE30: Major adverse kidney events within 30 days
MedDRA: Medical Dictionary for Regulatory Activities
MOO: Manual of Operations
NCATS: National Center for Advancing Translational Sciences
NIAID: National Institute of Allergy and Infectious Diseases
NIDDK: National Institute of Diabetes and Digestive and Kidney Diseases
NIH: National Institutes of Health
OR: Odds ratio
PECARN: Pediatric Emergency Care Applied Research Network
PEMCRC: Pediatric Emergency Medicine Collaborative Research Committee
PERC: Pediatric Emergency Research Canada
PHI: Protected health information
PI: Principal investigator
REB: Research Ethics Board
RRT: Renal replacement therapy
SAE: Serious adverse event
sIRB: Single Institutional Review Board
SOA: Schedule of activities
SOP: Standard operating procedure
STEC: Shiga toxin-producing *E. coli*
Stx: Shiga toxin
ULN: Upper limit normal
UP: Unanticipated problem
US: United States
